# The clinical value of indocyanine green fluorescence navigation system for laparoscopic partial nephrectomy in the case of complex renal clear cell carcinoma (R.E.N.A.L score ≥7)

**DOI:** 10.7150/jca.55033

**Published:** 2021-01-18

**Authors:** Rongjiang Wang, Jianer Tang, Yu Chen, Zhihai Fang, Junwen Shen

**Affiliations:** The first hospital of Huzhou, Zhejiang province, China.

**Keywords:** indocyanine green, fluorescence, partial nephrectomy, complex renal clear cell carcinoma

## Abstract

**Objective:** We demonstrated the potential clinical utility of the indocyanine green (ICG) fluorescence navigation system for laparoscopic partial nephrectomy in the case of complex renal clear cell carcinoma (R.E.N.A.L score ≥7).

**Methods:** Compared with the general laparoscopic partial nephrectomy and ICG fluorescence laparoscopic partial nephrectomy, a series of indicators were analyzed: the basic information like age, sex, and the tumor location; the operative information like the time of renal ischemia, the blood loss, and the complications; and other important indexes like the renal function, the volume of the tumor, and the weight of the specimens.

**Results:** 60 patients were included in this study. 21 patients in the group of fluorescence laparoscopy, and 39 patients in the group of general laparoscopy. There was no statistical difference for most indexes except the renal function. Preoperative serum creatinine was close (82.4±11.7 vs. 77.5±12.7, mmol/l, p=0.15). However, the patients in the group of fluorescence laparoscopy got a smaller serum creatinine growth degree (12.9±5.3 vs. 17.9±7.3, mmol/l, p=0.008), and a less decreasing level of GFR (16.5±6.4 vs. 24.4±9.8, mL/(min*1.73m^2^), p=0.001) after the operation. In addition, the average volume of the tumor (28.8±9.8 vs. 26.9±8.2, cm^3^, p=0.43) and the weight of the specimens (32.3±10.4 vs. 33.9±8.9, g, p=0.52) were no statistical difference. But the group of fluorescence laparoscopy had a smaller ratio of the weight/ the volume (1.13±0.06 vs. 1.28±0.10, g/cm^3^, p<0.001). And the two groups had a similar test-positivity rate of surgical margins (p=0.19).

**Conclusion:** Without increasing the rate of positive surgical margins, ICG fluorescence navigation system for laparoscopic partial nephrectomy for complex renal clear cell carcinoma could conserve more normal renal tissue.

## Introduction

With the remarkable update of surgical technology and equipment, the more complex renal tumors could undergo partial nephrectomy 1. Indocyanine green (ICG)-based fluorescence navigation system was one navel surgical technology 2. This technology was applied to the operation of partial nephrectomy in recent years 3. On the view of fluorescence mode, the renal clear cell carcinoma was dark gray. At the same time, the normal renal tissues looked bright. Based on the different views, the ICG fluorescence navigation system could help the surgeons to identify the renal clear cell carcinoma from the normal renal tissues 4, 5. With the help of the ICG-fluorescence navigation system, we guessed the surgeons could decrease the risk of tumor-free surgical margins and retain more renal tissue in the partial nephrectomy.

Compared with general laparoscopic partial nephrectomy, this study aimed to evaluate the clinical value of the ICG- fluorescence navigation system for laparoscopic partial nephrectomy in case of complex renal clear cell carcinoma (R.E.N.A.L score ≥7).

## Patients and methods

### Patient population

We researched, for this study, all the patients who did the laparoscopic partial nephrectomy in our institution from 2017 to 2019. The patients, who not only had been histopathological prognostic as renal clear cell carcinoma but also had a radiological diagnosis of a complex renal mass (R.E.N.A.L score ≥7), were rolled into our retrospective study. The patients were divided into two groups, according to different surgical modalities (the ICG fluorescence navigation system for laparoscopic partial nephrectomy or general laparoscopic partial nephrectomy). The surgical protocol strictly followed clinical practice guidelines 6, 7 and the consent was signed by patients or family members before the operation. This study got approval from our institutional ethics committee.

### Complex renal tumor

Preoperative imaging examinations for renal mass mainly included contrast-enhanced computed tomography (CT) within two weeks before the operation. The R.E.N.A.L nephelometry evaluation, based on the imaging of contrast-enhanced CT, was used for representing anatomical locations of renal mass. The tumor was considered complex renal neoplasm once the R.E.N.A.L score was greater than or equal to 7.

### General laparoscopic partial nephrectomy

Assessed by tumor size, location, and the relation of the tumor to vascular structures, the laparoscopic surgical approach contained trans- and retroperitoneal. Firstly, the renal pedicles were carefully isolated. Secondly, the surface of the renal tumor was exposed, and the border was identified carefully by the surgeons' eyes between the normal tissue and the tumor. Thirdly, renal ischemia was produced by renal artery occlusion using artery clips. A wedge resection of the tumor was performed with scissors. Absorbable sutures were used to close the renal incision. Fourthly, artery clips were loosened and renal color change was monitored to confirm blood reflow. In case prolonged bleeding persisted from the incision, further hemostasis was necessary by using a bipolar electric coagulator or staple the incision.

### The ICG- fluorescence navigation system for laparoscopic partial nephrectomy

Compared with general laparoscopy, the ICG fluorescence navigation system for laparoscopic partial nephrectomy needed to use special device displays and contrast agents. We made use of PINPOINT system laparoscopic camera and device displays, which contained not only the general mode of white light but also the fluorescence mode of green light, colorful light, black and white light. And the indocyanine green (ICG) was the necessary contrast agent. The surgical procedure was following. Firstly, the renal pedicles were carefully isolated. Secondly, the surface of the renal tumor was exposed. Thirdly, approximately 20-30 seconds after intravenous injection of ICG (1 ml), the renal artery was occluded by artery clips. In the fluorescence mode, the renal tumor (only for renal clear cell carcinoma) was darker than the surrounding normal tissue. And the border was easily and immediately recognized in the light of green or black and white. The whole wedge resection of the tumor was performed under the fluorescence mode of green light. After removing the renal tumor, three lights of fluorescence mode were alternately used to ensure complete tumor resection. In the end, the renal incision was close and artery clips were loosened.

### Data collected

To evaluate the surgical efficacy and complications of two different operations, the following data were collected. 1) Basic information: age, sex, and body mass index; 2) Tumor anatomical location: the maximum tumor diameter from contrast-enhanced CT imaging, tumor side, tumor location, and the complexity of tumors according to the R.E.N.A.L nephelometry score; 3) The degree of malignancy: the TNM stage; 4) The information of the operation: the operative time, the time of renal ischemia, the volume of the tumor which was measured in fresh specimens, the weight of the whole fresh specimens, and the weight/the volume of fresh specimens was calculated; 5) Blood loss: the blood loss during the operation, preoperative hemoglobin, and hemoglobin within 72 hours postoperatively. ΔHemoglobin was calculated subtracting postoperative hemoglobin from preoperative hemoglobin; 6) Renal function estimation: serum creatinine preoperatively, serum creatinine within 72 hours postoperatively, the lever of Glomerular Filtration Rate (GFR) preoperatively, and the lever of GFR within 72 hours postoperatively. We used the Modification of Diet in Renal Disease (MDRD) formula to assess the level of GFR. ΔSerum creatinine was calculated subtracting preoperative serum creatinine from postoperative serum creatinine. In the same way, ΔGFR was also calculated. 7) The complication: perioperative complications, postoperative complications as classified according to the Clavien system. 8) The prognostic information: the number of surgical margins, and the number of recurrences in the sixth month initial postoperatively. The patients were required to do the contrast-enhanced CT imaging of kidney and lung six months after the operation.

### Statistical analysis

SPSS25.0 statistical software was used to process the data, and the T-test was adopted for calculation data comparison and χ^2^ test for enumeration data comparison, where P<0.05 was defined as a statistically significant difference.

## Results

Ultimately, 60 patients were identified and retrospectively analyzed. 21 patients underwent the fluorescence laparoscopic partial nephrectomy, while the rest 39 patients were performed the general laparoscopic partial nephrectomy. All 60 patients were performed by two experienced surgeons. There were no differences in baseline variables between the two groups (**Table 1**).

All the enrolled patients had complex renal mass (R.E.N.A.L score ≥7), and the difficulty of the surgery for two groups was very close. The R.E.N.A.L score for the group of fluorescence laparoscopy was 7.9±0.8, while the R.E.N.A.L score for the group of general laparoscopy was 8.0±0.7 (P=0.56) (**Table 1**).

Focusing on the ischemia techniques, all the patients underwent global ischemia. The time of renal ischemia was 27.2±3.6 mins for the group of fluorescence laparoscopy, and 27.9±3.2 mins for the group of general laparoscopy (p=0.45) (**Table 2**).

The average blood loss for the group of fluorescence laparoscopy was 48.2±9.7 ml. In contrast, the average blood loss for the group of general laparoscopy was 47.4±7.1 ml (p=0.72). Compared with another index of hemoglobin variable between preoperation and postoperation, two groups still had similar results (13.5±4.8 vs 14.6±3.9, g/l, p=0.35).

By measuring the border of the tumor in the specimens, the length, the breadth, and the altitude of the tumor were recorded. The volume of the tumor was calculated (the volume =the length* the breadth* the altitude/2, cm^3^). The average volume of the tumor for the group of fluorescence laparoscopy was 28.8±9.8 cm^3^, while the group of general laparoscopy had a smaller average volume (26.9±8.2 cm^3^, p=0.43). At the same time, the specimen got weighed with an electronic weighing balance (the group of fluorescence laparoscopy: 32.3±10.4 g, the group of general laparoscopy: 33.9±8.9 g, p=0.52). However, the group of fluorescence laparoscopy had a smaller ratio of the weight/ the volume (1.13±0.06 vs. 1.28±0.10, g/cm^3^, p<0.001) (**Table 2**).

3 patients had surgical margins, and 2 patients had recurrences six months later in the group of general laparoscopy. In contrast, no patients had surgical margins or got recurrences in the group of fluorescence laparoscopy (**Table 3**).

4 patients had perioperative complications in the group of general laparoscopy: 3 patients had the urinary fistula, and 1 patient needed to do the artery selective embolization. While only 1 patient had a urinary fistula in the group of fluorescence laparoscopy. Clinical symptoms of urinary fistula for 4 patients got improved 1-3 months later. Because of repeated hematemesis after the operation, 1 patient in the group of general laparoscopy did the digestive endoscopy (Clavien ≥3). In comparison, no patient in the group of fluorescence laparoscopy had postoperative complications with Clavien ≥3 (**Table 2**).

Both of the preoperative serum creatinine and the lever of GFR were similar for two groups. But the patients in the group of fluorescence laparoscopy got a smaller serum creatinine growth degree (12.9±5.3 vs. 17.9±7.3, mmol/l, p=0.008), and a less decreasing level of GFR (16.5±6.4 vs. 24.4±9.8, mL/(min*1.73 m^2^), p=0.001) (**Table 3**).

## Discussion

For complex renal malignant tumors, partial nephrectomy still had several challenges and difficulties 8. Juan Garisto 9 had made an in-depth contrast between robot-assisted partial nephrectomy and traditional open partial nephrectomy. Specifically, his results suggested that a significant decrease in the estimated glomerular filtration rate was observed from the preoperative to postoperative period, regardless of the approach. Several studies 10, 11 revealed that various complications associated with complex renal tumors cannot be ignored. The risk of urine leakage, renal hematomas, and surgical margins increased significantly.

To significantly decrease the rate of postoperative recurrence, the thorough removal of tumors was important. This meant that some normal tissues inevitably needed to be resected in the process of the operation. While retaining residual renal function, we attempted to remove the normal renal tissue as little as possible. The balance cannot easily be resolved for the surgeons. With the rapid development of surgical instruments, navigational aids had been a new choice for surgeons 12, 13. At the time being, intraoperative ultrasound positioning had become widely used in clinical practice 14. Preoperative CT three-dimensional reconstruction may be a safe and effective procedure 15. However, the optimal surgical procedure for complex renal malignant tumors was currently unknown. From our experience, the ICG-based fluorescence navigation system could probably be the most suitable treatment.

Firstly, the ICG was combined with hemoglobin in the blood via intravenous injection. Secondly, ICG could be transported into major organs (for example, liver, kidney, spleen) because of the blood circulation 16. The sequential changes of ICG particle were transported into proximal tubule cells by such mechanisms as clearance by lymphatic drainage from the kidney after intravenous injection. This phenomenon likely explained why fluorescent signals were detectable in a normal kidney, where bilitranslocase functions in a pivotal role as a carrier protein of ICG 17. On the other hand, the loss of ICG-based fluorescence in a kidney tumor could be attributed to functional impairment of bilitranslocase in cancer tissue. Previous studies showed that the loss of ICG-based fluorescence only appeared in renal clear cell carcinoma 18, 19. Our clinical practice also presented that only renal clear cell carcinoma looked dark in the fluorescence mode. While other renal carcinomas, like eosinophilic renal carcinomas or papillary renal cell carcinoma, looked as bright as the normal renal tissues. We found 20-30 seconds after ICG intravenous injection might a perfect timing for renal ischemia. About 15-20 seconds after ICG intravenous injection, ICG by the passage of hemoglobin, traveled into the kidney (in the fluorescence mode, the renal arteries began to get development). 20-30 seconds after ICG intravenous injection, the renal vein began to develop, which meant that ICG was excreted primarily through the kidney. The largest degree of ICG was intensely detained in renal tissue if the renal artery was clamped at 20-30 seconds after ICG intravenous injection. A large variety of ICG in the kidney ensured that the normal renal tissue kept bright imaging and the clear border between the renal clear cell carcinoma and normal tissue.

Our study proved the value of the ICG-based fluorescence navigation system for laparoscopic partial nephrectomy in the case of complex renal clear cell carcinoma. Firstly, compared to general laparoscopic surgery, the fluorescence adjuvant laparoscopic surgery had a better postoperative renal function. The group of fluorescence laparoscopy had a more health serum creatinine and the lever of GFR. Secondly, the group of fluorescence laparoscopy had a smaller number of the weight/ the volume. The small index of the weight/ the volume represented that the fluorescence adjuvant laparoscopic surgery preserved a more physiological renal tissue. Thirdly, the fluorescence adjuvant laparoscopic surgery helped surgeons identify the border of the complex renal tumor and reduced the probability of positive surgical resection margins. However, there was no statistical difference for positive surgical margins between the two groups in our study.

## Conclusion

ICG fluorescence for laparoscopic partial nephrectomy could conserve more normal renal tissue without increasing the rate of positive surgical margins. In the complex renal clear cell carcinoma, ICG fluorescence for laparoscopic partial nephrectomy had trivial effect modification on renal function.

## Figures and Tables

**Figure 1:**
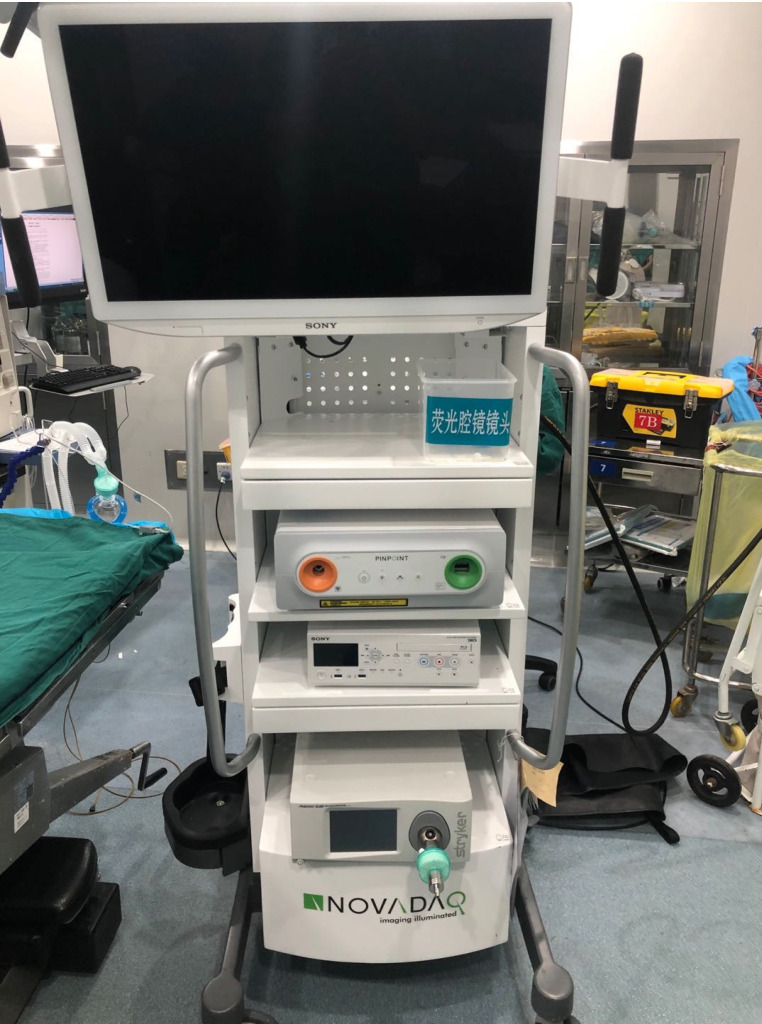
The vision of the PINPOINT system laparoscopic camera and device displays.

**Figure 2:**
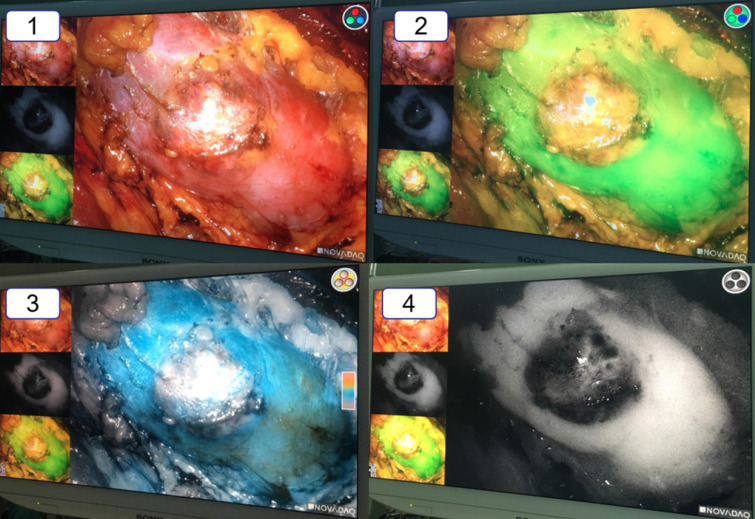
The vision of the ICG-fluorescence navigation system for laparoscopic partial nephrectomy. 1: The vision of the renal tumor in the white light. 2: The vision of the renal tumor in the green model. 3: The vision of the renal tumor in the color model. 4: The vision of the renal tumor in the black and white model. Compared with the normal renal tissue, the renal clear cell cancer looked obviously “darker”.

**Table 1:** The basic variables between the two groups

	The group of fluorescence laparoscopy (n=21)	The group of general laparoscopy (n=39)	*P* value
Age (year)	61.1±8.4	62.7±6.7	0.43
Sex (male/female, n)	11/10	20/19	0.94
Body mass index	23.3±1.9	23.9±2.0	0.27
**The tumor anatomical location**			
the maximum diameter in CT, cm	4.4±0.5	4.3±0.5	0.39
Anterior face/postior face, n	12/9	21/18	0.81
Tumor location: superior/intermediate/lower, n	8/8/5	8/15/16	0.25
Tumor growth: >50% exophytic, <50% exophytic, n	6/15	15/24	0.44
The distance between the tumor boundary and renal collecting ducts: >7 mm, 4-7 mm, <4 mm, n	2/12/7	3/17/19	0.24
The RENAL score	7.9±0.8	8.0±0.7	0.56

**Table 2:** The operative variables between the two groups

	The group of fluorescence laparoscopy (n=21)	The group of general laparoscopy (n=39)	*P* value
The operative time (min)	123.3±13.5	119.5±9.5	0.21
The time of renal ischemia (min)	27.2±3.6	27.9±3.2	0.45
The volume of the tumor (cm^3^)	28.8±9.8	26.9±8.2	0.43
The weight of the fresh specimens (g)	32.3±10.4	33.9±8.9	0.52
The weight/the volume (g/cm^3^)	1.13±0.06	1.28±0.10	<0.001
T stage: T1a/T1b, n	5/16	17/22	0.13
Blood loss (ml)	48.2±9.7	47.4±7.1	0.72
Preoperative hemoglobin (g/l)	138.2±7.5	138.3±6.0	0.93
ΔHemoglobin (g/l)	13.5±4.8	14.6±3.9	0.35
Perioperative complications, n	1	4	0.46
Postoperative complications according to the Clavien system: ≥3, <3, n	2/0	4/1	0.75

**Table 3:** The functional variables and prognostic information between the two groups

	The group of fluorescence laparoscopy (n=21)	The group of general laparoscopy (n=39)	P value
Preoperative serum creatinine (mmol/l)	82.4±11.7	77.5±12.7	0.15
Δ serum creatinine (mmol/l)	-12.9±5.3	-17.9±7.3	0.008
The lever of GFR preoperatively (mL/(min*1.73 m^2^))	106.6±20.1	114.1±20.1	0.17
Δ the lever of GFR (mL/(min*1.73 m^2^))	16.5±6.4	24.4±9.8	0.001
The number of surgical margins (n)	0	3	0.19
The number of recurrences (n)	0	2	0.29
